# Dorsal vagal complex and hypothalamic glia differentially respond to leptin and energy balance dysregulation

**DOI:** 10.1038/s41398-020-0767-0

**Published:** 2020-03-09

**Authors:** Lauren M. Stein, Rinzin Lhamo, Anh Cao, Jayme Workinger, Ian Tinsley, Robert P. Doyle, Harvey J. Grill, Gerlinda E. Hermann, Richard C. Rogers, Matthew R. Hayes

**Affiliations:** 1 Department of Psychiatry, University of Pennsylvania School of Medicine, Philadelphia, PA USA; 2 Department of Chemistry, Syracuse University, Syracuse, NY USA; 3 Institute of Diabetes, Obesity and Metabolism and Department of Psychology, University of Pennsylvania, Philadelphia, PA USA; 4 Department of Autonomic Neuroscience, Pennington Biomedical Research Center, Baton Rouge, LA USA

**Keywords:** Psychiatric disorders, Molecular neuroscience

## Abstract

Previous studies identify a role for hypothalamic glia in energy balance regulation; however, a narrow hypothalamic focus provides an incomplete understanding of how glia throughout the brain respond to and regulate energy homeostasis. We examined the responses of glia in the dorsal vagal complex (DVC) to the adipokine leptin and high fat diet-induced obesity. DVC astrocytes functionally express the leptin receptor; in vivo pharmacological studies suggest that DVC astrocytes partly mediate the anorectic effects of leptin in lean but not diet-induced obese rats. Ex vivo calcium imaging indicated that these changes were related to a lower proportion of leptin-responsive cells in the DVC of obese versus lean animals. Finally, we investigated DVC microglia and astroglia responses to leptin and energy balance dysregulation in vivo: obesity decreased DVC astrogliosis, whereas the absence of leptin signaling in Zucker rats was associated with extensive astrogliosis in the DVC and decreased hypothalamic micro- and astrogliosis. These data uncover a novel functional heterogeneity of astrocytes in different brain nuclei of relevance to leptin signaling and energy balance regulation.

## Introduction

The development of efficacious weight management therapies to treat obesity requires a broader understanding of the underlying biological basis of obesity. Current knowledge regarding the neural circuitry that regulates energy balance is limited, due in part to an incomplete characterization of the participation of non-neuronal cells such as glia. There are four known types of glia cells found within the central nervous system (CNS); astrocytes, oligodendrocytes, microglia, and ependymal cells. These glia cells represent an important potential target for the treatment of obesity and obesity-related disorders. Astrocytes are the most abundant glia cell type in the mammalian CNS^1^. Rather than acting as passive supporters of neuronal function, modern neuroscience widely acknowledges the dependence of neurons on astrocytes for synaptic efficiency and neuronal excitability (reviewed in ref. ^2^). Astrocytes participate in numerous CNS functions including assisting in the transport of circulating factors across the blood-brain barrier^3,4^, regulation of synaptic glutamate levels^5^ and the release of gliotransmitters^6^ that participate in neurotransmission (reviewed in ref. ^2^). Despite our growing appreciation of this bidirectional communication between astrocytes and neurons^7^, the specific, and likely heterogeneous, role of astrocytes in energy balance regulation is understudied.

To date, much of our knowledge about the roles of glia in the control of food intake and body weight regulation comes from seminal work in the hypothalamus. Indeed, hypothalamic astrocytes detect and transport nutrients^8–10^, aid in the development and plasticity of metabolic circuitry^11,12^ and respond to an array of neuropeptides and metabolic hormones such as the adipokine hormone leptin^13–15^. Some research hypothesizes that, as the resident immunocompetent cells of the brain^16^, microglia partially drive obesity-associated “neuroinflammation”. This hypothesis is supported by the observed association between the activation and proliferation of glia cells (defined as gliosis) in the hypothalamus and obesity-induced hypothalamic inflammatory cytokines (often overstated as “neuroinflammation”)^17–20^. Moreover, in rats susceptible to diet-induced obesity, there is significant hypothalamic inflammation^21^ and glia-mediated synaptic reorganization favoring orexigenic signaling^22^. Several studies further ascribe obesity-associated inflammation in the hypothalamus to alternations in leptin signaling at its cognate receptor (LepR). Indeed, leptin-deficient Ob/Ob mice exhibit profound hypothalamic gliosis when maintained on a high-fat diet^23,24^. The interactions of high-fat diet, leptin signaling, and neuroinflammation described in the medial hypothalamus and its relevance to energy balance regulation are largely uninvestigated in other key nuclei. Given that the neural control of energy balance is not restricted to the hypothalamic subnuclei, but rather is distributed throughout the brain^25,26^, an investigation of the role of glia in extra-hypothalamic nuclei governing energy balance may provide insight into the central dysregulation of diet-induced obesity, as well as the relationship between leptin and astrogliosis.

Looking beyond the hypothalamus, the dorsal vagal complex (DVC) of the caudal brainstem represents another CNS node in the detection, integration, and processing of numerous metabolic and endocrine signals, including leptin^27–29^. The DVC is comprised of the nucleus tractus solitarius (NTS), area postrema (AP), and dorsal motor nucleus of the vagus. Leptin receptor signaling in the DVC suppresses food intake and most notably potentiates the intake suppressive effects of within-meal gastrointestinally derived satiation signals (e.g., gastric distension and cholecystokinin)^28,30–34^. Despite a clear role for the DVC in energy homeostasis, very few studies characterize the specific cellular phenotypes that express LepR in the DVC. Evidence of LepR expression on NTS astrocytes^35^ led to the current hypothesis investigated here, that DVC glia are a prime cellular target for central leptin signaling. We investigated the expression of LepR on astrocytes in the DVC and assessed the role of DVC astrocytes in mediating the anorectic effects of leptin. Moreover, we examine the influence of obesity and perturbations in leptin signaling on the glia landscape in both the DVC and hypothalamus.

## Materials and methods

### Animals

Male Sprague Dawley rats (250–265 g, Charles River (UPenn) or Envigo (PBRC)) were maintained on either standard chow (Purina Rodent Chow 5001; Ralston Purina Company, St. Louis, Missouri) or a 60% high-fat diet (HFD; Rodent Diet D12492; Research Diets, New Brunswick, New Jersey) with ad libitum access to tap water. Zucker diabetic fatty rats (ZDF; Charles River) were maintained on a standard chow diet. Animals were single-housed in hanging wire cages in a temperature- and humidity-controlled environment on a reverse 12h:12h light-dark cycle. All behavioral experiments were carried out in a counter-balanced, within-subjects design, no randomization required. All experimental procedures were conducted with the approval of the University of Pennsylvania and Pennington Biomedical Research Center Institutional Animal Care and Use Committees.

### Stereotaxic surgeries

Rats were anesthetized with an intramuscular injection of ketamine (90 mg/kg; Butler Schein), xylazine (2.7 mg/kg; Midwest Veterinary Supply), and acepromazine (0.64 mg/kg; Midwest Veterinary Supply). Using stereotaxic surgery, rats were implanted with an indwelling cannula (26-gauge; Plastics One) directed at the 4th cerebroventricle (4V; coordinates: on midline, 2.5 mm anterior to the occipital suture and 7.2 mm ventral to the skull^36^. Postoperative analgesia (2 mg/kg meloxicam) was administered subcutaneously for 2 days, and animals were allowed to recover for one week. Proper placement and cannula patency were verified before behavioral testing via 5-thio-D-glucose (210 μg)-induced hyperglycemia as previously described^37^; only animals that passed verification were tested.

### Synthesis and administration of Cy5-labeled leptin

Recombinant mouse leptin (2.0 mg; NHPP) was dissolved in 500 μL of 50 mM phosphate buffer (pH 7.4) and gently rocked in non-stick Eppendorf tubes (Thermo Scientific, Cat no. 3451). A quantity of 0.2 mg sulfo-cyanine 5 NHS ester (Lumiprobe, Cat no. 23320) was dissolved in 50 μL DMSO and then added to the pre-dissolved leptin in 10-μL aliquots over 1 h. The reaction was stirred at ambient temperature overnight then purified using a Shimadzu Prominence HPLC with a C18 column (Eclipse XDB-C18 5μm, 4.6 x 150 mm) and a gradient of [1% CH_3_CN/H_2_O + 0.1% to TFA] to [70% CH_3_CN/H_2_O + 0.1% to TFA] over 15 min. The product was obtained with a ~T_R_ of 10 min at 98% purity, lyophilized, and stored at –20 °C. Electronic absorption spectra were obtained on a Varian Cary 50 Bio spectrophotometer in a 2 mL quartz cuvette between 500–800 nm in aqueous acetonitrile. An absorption maximum was identified at 651 nm. Fluorescence spectra were obtained on an Agilent Cary Eclipse Fluorescence Spectrophotometer in a 2 mL cuvette between 500–800 nm in aqueous acetonitrile. The instrument was used in fluorescence mode with the excitation slit at 5 nm and emission slit at 5 nm. Excitation occurred at 651 nm with an emission maximum at 669 nm. SDS-PAGE was performed using an Invitrogen Mini-cell GEL Surelock Cell Module with 12% tris-glycine gel in SDS running buffer. Protein masses were identified using the Precision Plus Protein Kaleidoscope pre-stained protein standard (BioRad, Cat no. 1610375) (Supplementary Fig. 1). A standard chow-fed male rat with an indwelling 4V cannula received a single icv injection of 4.5 ng (3 μL at 1.5 ng/mL) fluorescently labeled leptin (Cy5-Lep) dissolved in 0.1 M PBS, pH 7.4. An hour later, the rat was anesthetized, transcardially perfused, and harvested for brain removal and IHC processing as described below. Images acquired with Leica SP5 X confocal microscope using 63x oil-immersion objective with 405, 488, 647 laser lines. Image z-stacks were collected with a step size of 1μm and processed using Imaris 8.1.2 software (Bitplane).

### Fluorescent in situ hybridization of astrocytic LepR

For verification of the leptin receptor on rat DVC astrocytes, a single animal (*n* = 1) was anesthetized for rapid removal of the brain and flash frozen in isopentane over dry ice. The brain was sectioned on a cryostat at 18 μm thickness, slide mounted, and stored at −80 °C. Fluorescent in situ hybridization (FISH) was performed using the RNAscope Multiplex Fluorescent Reagent kit v2 (Cat no. 323100; ACDBio, Hayward, CA) per manufacturer instructions. Detection was carried out using probes designed by ACDBio for LepR mRNA (Rn-LepR-C1, Cat no. 415951), the astrocyte-specific marker Aldh1L1 (Rn-Aldh1L1-C2, Cat no. 459821-C2), and the neuronal marker RbFox3 (Rn-RbFox3-C3, Cat no. 436351-C3). Following a series of amplification steps, sections were mounted with DAPI-containing mounting media (Fluorogel; Fischer Scientific). Sections were visualized with a Leica SP5 X confocal microscope with a 40x objective. Image z-stacks were collected with a step size of 1 μm. Collected z-stack images were processed using Imaris 8.1.2 software (Bitplane).

### Live cell Ca^+2^ imaging of neurons and astrocytes in NTS slice preparations

Male Sprague Dawley rats (290–420 g; chow *n* = 4, HFD *n* = 3) were deeply anesthetized with urethane (1.5 g/kg, intraperitoneal; ethyl carbamate, Sigma; this anesthetic was selected because it readily washes out of ex vivo slices^38^) and placed in a stereotaxic frame. Using aseptic technique, 4 unilateral injections (40 nL each) of 0.4% Cal520 (AAT Bioquest), 0.3% sulforhodamine 101 (SR101; Sigma Chemical) and 10% pluronic-DMSO (F-127, Invitrogen) in normal Krebs solution were administered into the medial NTS. After 60 min, rats were decapitated, and the brainstem was quickly harvested. Pre-labeled brainstems were cut into 300-μm coronal sections using a vibrating microtome (Leica VT1200) and submerged in cold (~4 °C) carboxygenated (95% O_2_; 5% CO_2_) cutting solution. NTS slices were placed in normal Krebs’ solution and bubbled with 95% O_2_/5% CO_2_ at a constant temperature of 29 °C. Sections were allowed equilibrate for 1 h prior to imaging^39^.

Live cell calcium imaging of pre-labeled neurons and astrocytes was performed as previously described^39–41^. Cal-520/SR101 pre-labeled slices were transferred to a custom imaging chamber^42^. Immunohistochemical confirmation of SR101 specificity to astrocytes in the DVC was previously reported^43^. The recording chamber was continuously perfused at a rate of 2.5 mL/min with normal Krebs’ (or containing 100ng/mL leptin) solution at a constant temperature of 33 °C. Hindbrain slices were viewed with a Zeiss Axioskop 2 fixed stage microscope equipped with normal epifluorescence optics, a Yokogawa CSU21 laser confocal scan head, and a Hamamatsu ORCA-ER camera. Pre-labeled cells of interest were selected visually with epifluorescence optics and subsequently confocal images were captured with the ORCA-ER camera at the rate of 1 frame per second.

Once in the recording chamber, hindbrain slices were perfused with normal Krebs’ solution for a minimum of 10 min. Dual exposure images (i.e., 488 nm and 591 nm) were collected just prior to each experimental trial in order to confirm the cell types being recorded. All slices were then challenged with a cocktail of 100 μM ATP and 500 μM glutamate to determine which cells in the field were viable^39,44^. Viability was defined as a minimum increase in fluorescence of 7% in response to the ATP/glutamate challenge^45^. Next, slices were exposed to Krebs’ solution plus 100 ng/mL leptin for 30 s. Changes in intracellular calcium concentrations in response to leptin stimuli within Cal-520 pre-labeled NST astrocytes and neurons were recorded simultaneously using the 488-nm laser line to excite the Cal-520. Increases in intracellular calcium concentrations were identified as an increase in fluorescence and interpreted to represent increased cellular activity. During experimental trials, time-lapse images of mixed fields of NTS astrocytes and neurons were monitored for responses to the leptin challenge. Time-lapse laser confocal images of changes in intracellular calcium levels of both astrocytes and neurons were captured with the ORCA-ER at a rate of 1 frame per second.

### Behavioral experiments

Rats were maintained on either standard chow (*n* = 10) or a high-fat diet for 2 weeks (*n* = 7). All animals were implanted with a cannula directed at the 4th ventricle (4V) to allow for intracerebroventricular (icv) administration. The recovery period was 1 week, during which time placement of the cannula was verified with a hyperglycemic response to 5-TG^46,47^. Food hoppers were removed, and animals were weighed approximately 1 h before dark onset. Thirty min before dark onset, animals received 4V icv vehicle (0.1 M PBS) or 50 nmol fluorocitrate pretreatment (Sigma Aldrich^40,48–50^) followed by vehicle (0.1 M NaHCO3) or 5 μg/mL leptin (NHPP^51^). Fluorocitrate is a well-established pharmacologic agent used to transiently inhibit the Kreb’s cycle in an astrocyte-specific manner^48,50,52–54^. The selected dose of 50nmol fluorocitrate is established to be astrocyte-specific^40,48^. At dark onset, food hoppers were replaced and cumulative food intake was recorded at 1, 3, 6, and 24 h. Body weights were recorded at 0 and 24 h after injection. Experimental injections were separated by a 72 h washout period.

### Immunohistochemical analysis of gliosis

Rats were placed on either a standard chow (*n* = 6) or a high-fat diet (*n* = 6) for 8 weeks. Food intake and body weights were recorded every 48 h. After the 8-week period, anesthetized animals were transcardially perfused with 0.1 M sodium phosphate buffer (PBS; pH 7.4) followed by 4% paraformaldehyde (4% PFA) in 0.1 M PBS, pH 7.4. Brains were removed and stored in 4% PFA overnight at 4 °C, then transferred to a 20% sucrose solution in 0.1 M PBS. Brains were flash frozen in hexane over dry ice. Subsequently, the DVC and hypothalamus were sectioned coronally at 30 μm. Free-floating serial sections were processed for immunohistochemical detection of glia fibrillary acidic protein (GFAP; astrocyte activation marker^18,55^) and ionized calcium binding adaptor molecule 1 (Iba1; microglia marker^56^) as follows. Sections were washed 3 times for 5 min each in 0.1 M PBS, incubated in 10 mM sodium citrate buffer solution (Sigma Aldrich) for 30 min at 90 °C, then washed an additional 3 times in 0.1 M PBS containing 0.2% Triton-X (Sigma Aldrich). Next, sections were incubated in 5% normal donkey serum (NDS; Jackson ImmunoResearch) and 0.2% Triton-X in 0.1 M PBS for 1 h at RT, then incubated overnight at 4 °C in an antibody mixture of 1:1000 chicken anti-GFAP (Millipore, Cat no. AB5541) and 1:500 rabbit anti-Iba1 (Wako; Cat no. 019-19741) in blocking buffer. Following wash steps, sections were incubated for 2 h in secondary antibody mixture of 1:500 donkey anti-chicken Alexa 594 (Jackson ImmunoResearch) and 1:500 donkey anti-rabbit Alexa 488 (Jackson ImmunoResearch). Sections were mounted to Superfrost Plus slides (Fisher Scientific) with Fluorogel DAPI-containing mounting media (Fischer Scientific). Images were acquired using a Keyence BZ-X800 fluorescent microscope under a 20x or 40x objective as 1-μm z-stack images. Z-stack images were compressed using standard full focus and background subtraction. During image capture and analysis, investigator was blinded regarding corresponding diet cohort. Quantification was done using FIJI software. The integrated density of GFAP immunoreactivity was determined after default thresholding of images^57^. Microglia density was determined using the MorphoLibJ plugin^58^.

### Statistics

All data are represented as the mean ± standard error of the mean. Power analysis was conducted prior to carrying out experiments using an alpha of 0.05 and 80% power. Comparisons in qPCR studies were made using a 1-way analysis of variance (ANOVA) with Dunnet post hoc tests. For live calcium imaging, significance was determined by one-way ANOVA with Bonferroni t-tests. Comparisons of percentage of responsive cells were performed using Fisher’s exact test. For behavioral comparisons of food intake and body weight, we applied a repeated measure 3- or 2-way ANOVA, respectively, with Neuman-Keuls post hoc analyses. Statistical significance was set at *p* < 0.05. Statistical outliers were identified using the ROUT method (*Q* = 0.5%) and excluded from analysis.

## Results

### DVC astrocytes express the leptin receptor

**Fig. 1:**
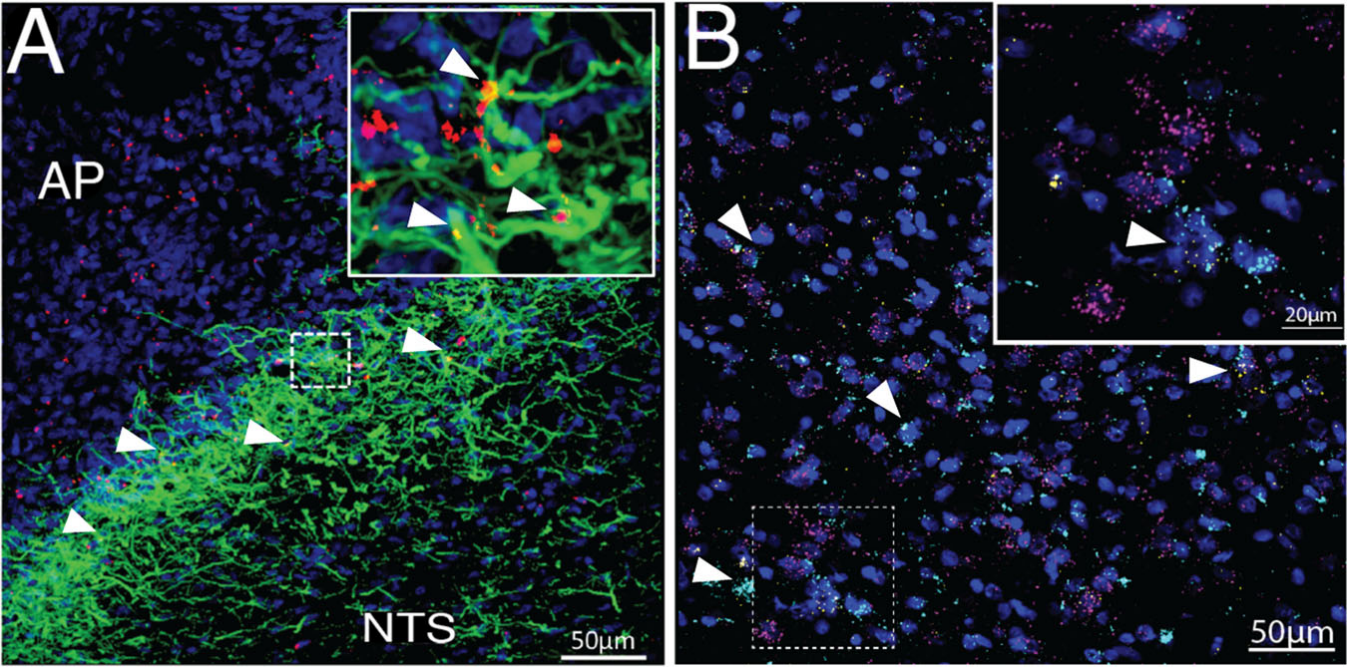
Immunohistochemical and FISH validation of leptin binding and LEP-R expression on DVC astrocytes. **a** Immunohistochemical visualization of Cy5-Lep (4V icv 4.5 ng; red) colocalized to DVC astrocytes (GFAP; green), DAPI in blue; 63x image (inset: 2x optical zoom). **b** FISH visualization of the lateral NTS (at the level of the AP) of LEP-Rb (yellow) expression on DVC astrocytes (ALDH1L1; cyan) and neurons (RbFox3; magenta). Counterstain with DAPI (blue); 40x image (inset: 3.4x optical zoom with gaussian filter). See Supplementary Video 1 for 3D rotational image of area outlined by the dotted box.

The expression of the leptin receptor on hypothalamic astrocytes is well established in rats and mice^14^^,15,35,59^. To complement, we performed immunohistochemistry to confirm LepR expression on DVC astrocytes in the rat. Following 4^th^ intracerebroventricle (4V) injection of fluorescently labeled leptin (4.5 ng Cy5-Lep), we observed co-localization with DVC astrocytes (GFAP; green; Fig. 1a), predominately at the subpostrema, the border of the AP and NTS. The presence of LepR expression on rat DVC astrocytes was detected by FISH using RNA-scope. LepR mRNA expression (yellow) was observed on both neurons (magenta) and astrocytes (cyan) within the subpostrema and medial NTS (Fig. 1b; Supplementary Video 1).

### High fat diet-induced obesity reduces the percentage of both DVC astrocytes and neurons showing leptin-induced Ca^+2^ signaling in ex vivo brainstem slice preparations

**Fig. 2:**
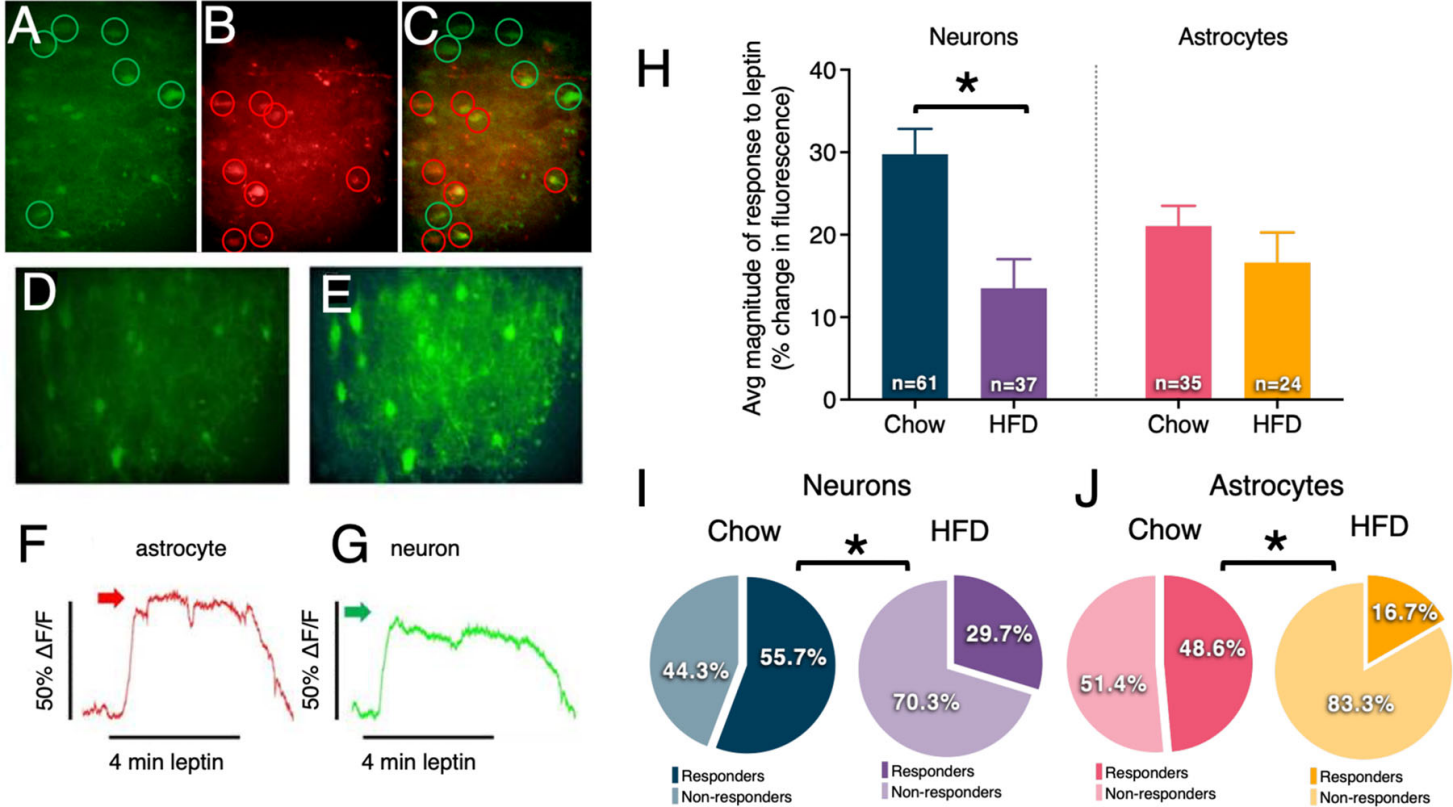
Leptin-induced Ca^+2^ flux in DVC neurons and astrocytes vary by dietary status. Live cell calcium recordings in DVC brain slices in astrocytes and neurons simultaneously. **a** Cells prelabeled with Cal520 (calcium sensitive dye; taken up by both neurons and astrocytes) encircled in green. **b** Cells prelabeled with SR101 (astrocyte-specific dye) are identified as astrocytes and encircled in red. **c** Yellow localization confirms that the calcium-sensitive dye is localized within astrocytes and distinguishable from neurons (green circle). **d** Total field of view (showing both neurons and astrocytes) at “rest” (i.e., baseline) and the same field of view shown in **e** at period of peak response leptin perfusion (see Supplementary Video 2 and 3). **f** Response to leptin perfusion of individual astrocyte marked by red arrow in **g** Response to leptin perfusion of individual neuron marked by green arrow in panel. Changes in fluorescence were measured in brain slices prepared from lean rats (*n* = 4) and rats exposed to a high-fat diet (*n* = 3) following bath leptin exposure (100ng/mL). **h** Magnitude of the leptin response was attenuated in neurons but not astrocytes of obese compared to lean animals. Obese animals had lower proportions of leptin-responsive (**i**) neurons and (**j**) astrocytes compared to lean animals. Changes in magnitude are represented as the mean ± standard error of the mean and were analyzed with a 2-way ANOVA and Bonferroni post hoc tests; *p < 0.05. Percentages of leptin-responsive cells were analyzed using Fisher’s exact test; *p < 0.05. See Supplementary Video 2 and Supplementary Video 3.

To better characterize the role of DVC astrocytes in energy homeostasis, we next investigated leptin-induced intracellular Ca^+2^ responses in DVC neurons and astrocytes in ex vivo brainstem slice preparations (Fig. 2). In addition, we examined whether leptin-induced responses were altered by dietary status and obesity (i.e., comparison of Ca^+2^ signaling in the DVC of lean rats maintained on a standard chow or obese rats maintained on a 60% high-fat diet). Imaging experiments on the pre-labeled hindbrain slices from chow-maintained rats (*n* = 4) yielded 61 viable neurons and 35 viable astrocytes; high fat diet-maintained rats (*n* = 3) yielded 37 viable neurons and 24 viable astrocytes.

Both neurons and astrocytes from lean animals produced live cell Ca^+2^ flux (i.e., increases in cytoplasmic Ca^+2^) in response to bath application of 100ng/mL leptin (Fig. 2h). High-fat diet maintenance attenuated DVC neuron Ca^+2^ response without affecting astrocyte responses (*F*_3,62_ = 4.072; *p* = 0.01). Further, animals exposed to a high-fat diet had a significant reduction in the percentage of leptin-responsive neurons and astrocytes compared to lean animals (Fig. 2i, j). In astrocytes, this suggests a potential alteration in functional expression of LepR rather than changes in leptin responsiveness at the cellular level. Representative videos show a mixed neuro-astrocytic field of live calcium imaging following leptin administration in chow (Supplementary Video 2) and HFD (Supplementary Video 3) maintained rats.

### DVC astrocytes partly mediate the anorectic effects of leptin in lean but not obese rats

**Fig. 3:**
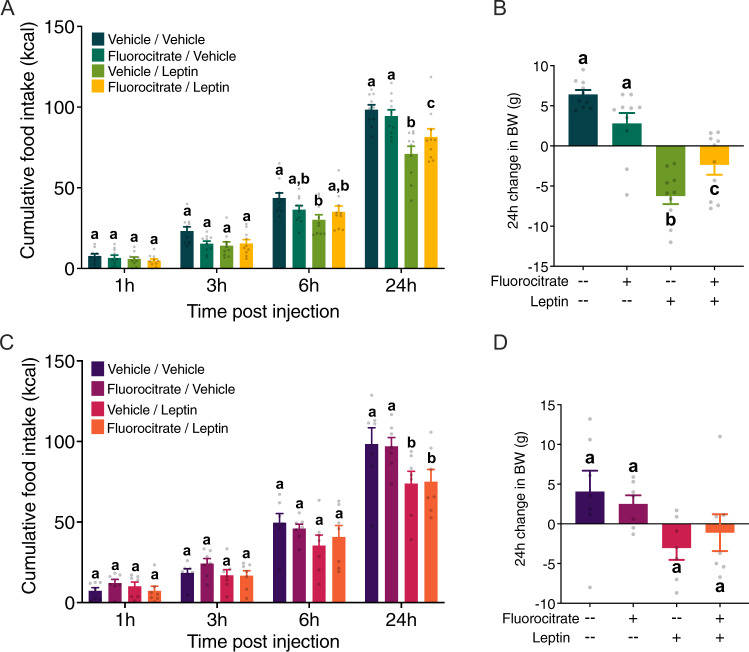
DVC astrocytes partly mediate the anorectic effects of leptin in lean but not obese rats. Changes in cumulative food intake (**a**, **c**) and 24-h body weight (**b**, **d**) were recorded following 4V icv pretreatment with 50 nmol fluorocitrate and subsequent treatment with 5 μg/mL leptin in rats maintained on either standard chow (*n* = 10; **a**, **b**) or a high-fat diet (*n* = 7; **c**, **d**). Data were analyzed using a 3-way (**a**, **c**) or 2-way (**b**, **d**) repeated measures ANOVA. Lowercase letters indicate significant differences (*p* < 0.05) within each time point (**a**, **c**) for food intake and at 24 h (**b**, **d**) for body weight according to Neuman-Keuls post hoc analyses.

A well-established pharmacologic approach was used to assess the contribution of DVC astrocytes in mediating the intake and body weight suppressive effects of leptin. Food intake (Fig. 3a, c) and consequent body weight change (Fig. 3b, d) were measured in rats following 4V administration of 50 nmol fluorocitrate, an astrocyte-specific Krebs cycle inhibitor^48,52–54^, and 5 μg/mL leptin^51^. At the time of experimental treatment, animals fed a high-fat diet weighed significantly more than chow-maintained animals (Supplementary Fig. 2). While there appears to be a slight trend, fluorocitrate treatment alone did not significantly affect food intake or body weight change in either group. In lean rats, pretreatment with fluorocitrate modestly attenuated the food intake inhibitory effects of leptin on 24-h food intake (fluorocitrate × leptin interaction: *F*_1,9_ = 7.678, *p* = 0.022; fluorocitrate x leptin x time interaction: *F*_3,27_ = 2.677; *p* = 0.067; Fig. 3a) and body weight gain (fluorocitrate x leptin interaction: *F*_1,9_ = 11.19; *p* = 0.009; Fig. 3b). Conversely, leptin administration in early onset obese rats exerted a modest hypophagic effect at 24hr (fluorocitrate × leptin interaction: *F*_1,6_ = 21.61; *p* = 0.009; *F*_3,18_ = 0.899, *p* = 0.46; Fig. 3c) and did not alter 24h body weight change (fluorocitrate × leptin interaction: *F*_1,6_ = 1.348, *p* = 0.29; Fig. 3d).

### Maintenance on a high-fat diet decreases DVC astrocyte activation

**Fig. 4:**
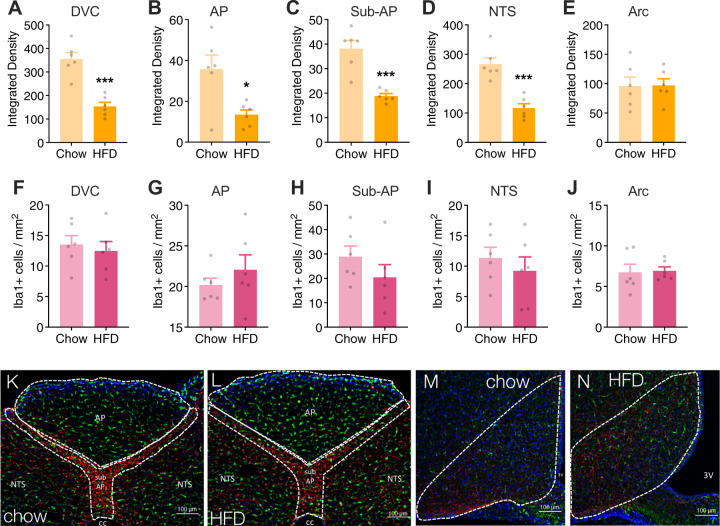
A high-fat diet alters gliosis in the brainstem but not the hypothalamus. Astrogliosis and microglial infiltration determined by the degree of GFAP immunoreactivity (**a**–**e**) and density of Iba1 positive cells (**f**–**j**), respectively. HFD significantly decreased GFAP immunoreactivity but did not affect microglial density. Diet had no influence on the glial landscape of the arcuate nucleus. Representative images (20x) of the DVC of lean (**k**) and obese rats (**l**) and of the hypothalamus of lean (**m**) and obese rats (**n**). Images were quantified in ImageJ and analyzed using unpaired *t*-tests; *p < 0.05, **p < 0.01, ***p < 0.001 compared to the lean group (*n* = 6 per group). AP, area postrema; NTS, nucleus tractus solitarius; sub-AP, sub-area postrema; cc, central canal; 3V, third ventricle.

Maintenance on a high-fat diet is associated with increased glia activation and proliferation in the hypothalamus^17,18,23^, such that the degree of astrogliosis may predict metabolic outcomes^60^. Given the observation that a high-fat diet was associated with fewer leptin-responsive neurons and astrocytes in the DVC (Fig. 2) and an attenuated hindbrain leptin response in vivo (Fig. 3), we next investigated maladaptive changes in the neuro-glia landscape of the DVC in response to obesity and, within the same rats, compared these changes to those observed in the arcuate nucleus of the hypothalamus. Rats that were fed either standard chow or a high-fat diet for eight weeks were sacrificed for immunohistochemical analysis of astrocyte activation (measured by GFAP integrated density^18,55^) and microglia infiltration (measured by density of Iba1+ cells^56^) in the whole DVC and individual nuclei including the AP, sub-AP, NTS, as well as the arcuate nucleus of the hypothalamus. Rats fed a high-fat diet had significantly lower GFAP integrated density in the entire DVC (Fig. 4a) as well as in all distinct nuclei of the DVC (Fig. 4b–d) compared to animals fed standard chow. There was no effect of diet on microglia density in the DVC (Fig. 4f–i). There was no detectable effect of a high-fat diet on gliosis within the arcuate nucleus (Fig. 4e, j).

### Loss of LepR signaling promotes astrogliosis in the DVC

**Fig. 5:**
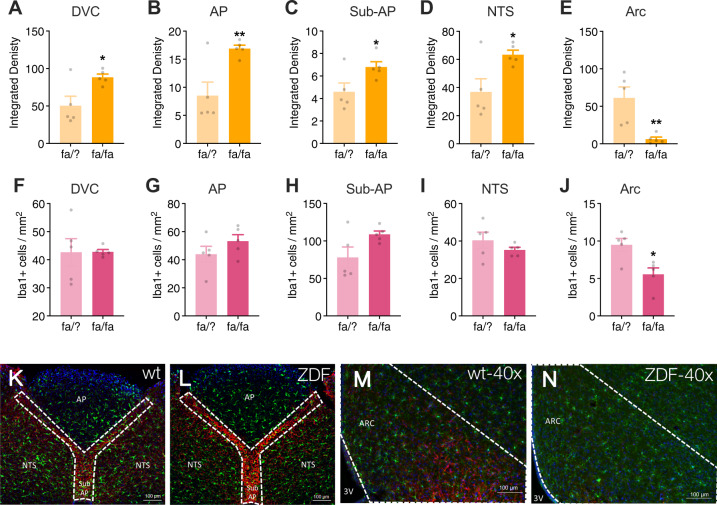
Zucker diabetic fatty rats (ZDF) exhibit heightened astrogliosis in the DVC and attenuated gliosis in the arcuate nucleus compared to wild type (WT) rats. Astrogliosis was determined by quantifying the integrated density of GFAP immunoreactivity within distinct regions of the DVC. ZDF rats had significantly higher GFAP integrated density in the DVC (**a**), AP (**b**), sub-AP(**c**), and NTS (**d**) compared to WT rats. There was no difference in microglial density in the DVC between ZDF and WT rats (**f**–**i**). Conversely, there was a significant reduction in both GFAP integrated density (**e**) and microglial density (**j**) in the arcuate nucleus of ZDF vs. WT rats. Representative images at 20x magnification of WT (**k**) and ZDF (**l**) dorsal vagal complex and 40x magnification of WT (**m**) and ZDF (**n**) arcuate nucleus. Images were quantified in ImageJ and analyzed using unpaired *t*-tests; *p < 0.05, **p < 0.01 compared to the WT group (*n* = 5 per genotype). AP, area postrema; NTS, nucleus tractus solitarius; sub-AP, sub-area postrema; cc, central canal; 3V, third ventricle; Arc, arcuate nucleus.

Zucker diabetic fatty rats (ZDF) have a mutation within the leptin receptor that prevents downstream signaling following leptin binding^61,62^. This form of leptin receptor deficiency results in obesity, hyperphagia, and hyperglycemia (Supplementary Fig. 3). Obesity due to deficient leptin receptor signaling in these ZDF rats was associated with significantly higher astrogliosis in the DVC (Fig. 5a–d) but a marked reduction in density of GFAP-positive cells in the arcuate nucleus compared to wild-type control rats (Fig. 5e). Microglia density was significantly lower in the arcuate nucleus of ZDF rats compared to wild-type rats (Fig. 5j). In contrast, no alteration in microglia density was observed in the DVC of ZDF rats compared to control rats (Fig. 5f–i).

The neurobiological control of energy balance involves a vast, interconnected network of neuronal crosstalk that continually senses and responds to an organism’s metabolic status^25,26^. Recent work has highlighted a role of glia in energy balance, but our limited knowledge regarding glia-neuron interplay in brain regions beyond the arcuate hypothalamus necessitates further exploration^48^. Here, we examined the contribution of caudal brainstem DVC astrocytes to the effects of leptin on food intake, as well as the effects of diet on glia cell density and activation in the DVC. Similar to previous characterizations of hypothalamic astrocytes^13,14,63^, an important main finding of our research was the discovery that DVC astrocytes are an additional site for leptin receptor expression and activation. The IHC and in situ observations combined with ex vivo live cell recordings of astrocytes demonstrate functional LepR receptors on rodent DVC astrocytes. Astrocyte-specific inhibition revealed a functionally relevant role of hindbrain astrocytes in mediating the effects of leptin administered into the 4^th^ ventricle. The current data also points to the DVC astrocytes as a site of leptin dysfunction in obesity. Calcium imaging revealed that animals fed a high-fat diet also exhibited a reduction in the proportion of leptin-responsive neurons and astrocytes in the DVC compared to lean animals. Exposure to a high-fat diet elicited the selective *reduction* of astrogliosis in the DVC, in contrast with previous studies of the hypothalamus^17–19^, and no effect on arcuate hypothalamic astrocytes. Further, the absence of leptin signaling in Zucker diabetic fatty rats was associated with enhanced astrogliosis in the DVC and decreased hypothalamic astrogliosis. These data in no way suggest that the DVC astrocytes are the only cellular substrate for leptin signaling or obesity-induced leptin resistance. Instead, our data highlight the DVC as a critical and overlooked CNS cellular site-of-action that may be targeted for restoring leptin signaling in obesity.

The role of hypothalamic astrocyte LepR signaling in energy homeostasis is well established in previous studies: knockdown of LepR in the hypothalamus leads to alterations in neural circuitry and attenuates leptin-induced anorexia in mice^15,14,64^. Yet, the significance of astrocytic LepR expression in the DVC is unknown. For this reason, we performed a series of pharmacologic experiments to estimate the involvement of the DVC astrocytes in the effects of leptin on food intake. We observed effects of 4V leptin on overnight food intake and subsequent weight gain in lean animals and further found that these effects were modestly attenuated by pretreatment with 4V fluorocitrate, an astrocyte-specific inhibitor of cellular respiration^48,50,52,53^. Albeit not significant, there appeared to be a slight inhibitory trend of fluorocitrate alone on food intake. As astrocytes and neurons comprise the tripartite synapse in which surrounding glia cells form intimate association with pre- and post-synaptic membranes^7,65,66^, it is plausible that a transient non-specific inhibition of astrocytes with fluorocitrate potentially elicits a modulation of vagal-NTS signaling to transiently decrease food intake. Interestingly, the attenuating effect of 4V fluorocitrate was absent in rats fed a high-fat diet. As the duration of HFD exposure does not correspond with a full manifestation of leptin resistance^67,68^, these data indicate a surprising, albeit limited role for hindbrain astrocytes in leptin responsiveness in obesity, and moreover, suggest that a loss of leptin responsiveness in hindbrain astrocytes represents part of the maladaptive neuroregulatory response to obesity in rodents. This idea is consistent with numerous reports describing a causal association between HFD and hypothalamic leptin resistance^11,17–19,21^. Our calcium imaging results support this statement, in which exposure to a high-fat diet significantly reduced the proportion of leptin-responsive astrocytes and neurons and decreased the magnitude of neuronal (but not astrocytic) responses to leptin in the DVC. While we speculate this may be a result of maladaptive changes to LepR functional expression, one cannot rule out the potential for alternative LepR signaling cascades within astrocytes. Indeed, Yasumoto et al. provide evidence of preference toward leptin- mediated increases in extracellular receptor kinase (ERK) expression as opposed to the classical increase in phosphorylation of STAT3^69^. Therefore, in order to provide mechanistic insight into leptin action on astrocytes and the effect of diet on astrocytic LepR signaling is required.

Astrocytes serve as the predominant regulator of synaptic glutamate (reviewed in ref. ^70^) Given that vagal afferent transmission of all satiation signaling from the GI tract is glutamatergic, it is intriguing to consider what effect leptin-astrocyte signaling would have on modulating vagal-to-NTS glutamatergic signaling. Leptin administration into the lateral ventricle results in a significant decrease in astrocyte specific glutamate transporters, GLT1 and GLAST, in the hypothalamus^63^. Thus, in the lean state, the well documented ability of DVC leptin signaling to suppress food intake via enhancement of GI-derived satiation signals^31,71–73^, may actually involve a leptin-mediated astrocyte specific suppression in astrocyte glutamate re-uptake transporters and subsequent augmentation of vagal glutamatergic transmission. A logical extension of this hypothesis would therefore follow that in the HFD-induced obese state, leptin’s ability to downregulate these astrocyte glutamate transporters would be blunted, consistent with our observed decrease in astrocyte Ca^++^ responsivity to leptin in HFD NTS slice preparations. It is clear that further investigations are warranted to assess the evolving role of DVC astrocytes in mediating leptin signaling and modulation of vagal-to-NTS glutamatergic transmission.

Another major finding of the current data, when considered in the context of pre-existing literature on the hypothalamus, was the remarkable divergence in the responses of DVC and hypothalamic glia cells to obesity. Since microglia serve as the resident immunocompetent cells of the central nervous system^74^ and previously shown to be responsive to high fat diet and obesity, we measured the effects of HFD and absence of leptin signaling on microglia density. Exposure to a high fat diet alone did not result in changes to microglia density in either the DVC or hypothalamus. Future characterization of microglia state, i.e., resting vs. activated, is required to determine whether diet and/or leptin increase the number of activated microglia.

Taken together, these findings support the hypothesis that DVC astrocytes contribute to energy balance regulation and constitute a component of the maladaptive changes that occur in response to diet-induced obesity. Moreover, our results suggest that astrocytes in the DVC may be particularly susceptible to the loss of leptin signaling.

Finally, we performed an in-detail characterization of gliosis in the DVC and hypothalamus of the same rats in order to better characterize the interplay of DVC astrogliosis and leptin signaling in response to a high-fat diet. Our observation that fluorocitrate attenuated leptin responses in lean but not obese animals indicated the possibility of corresponding astrocytic changes at the level of the brainstem. To test this hypothesis, we quantified gliosis (astrocytic and microglia) in specific areas of the DVC and hypothalamus in lean rats, rats exposed to a high-fat diet, and ZDF rats. Both HFD-induced obesity and ZDF rats serve as models of obesity; both are hyperphagic, hyperglycemic and increased adiposity. The Zucker diabetic fatty rat (ZDF) is an inbred sub-strain of the obese Zucker fatty rat, with a missense mutation in the LepR gene resulting in non-functional leptin signaling^62^. ZDF rats exhibiting early hyperglycemia, especially when maintained on a high fat diet. The HFD-induced obese model serves more clinical relevance as most forms of human obesity are due to polygenic alterations and environmental factors. Chronic exposure to a HFD not only results in an obese phenotype but is accompanied by the gradual development of leptin resistance. Studies in both humans and rodents revealed that at the level of the hypothalamus there is significant gliosis as a result of HFD exposure. Utilization of these two models begins to disentangle driving forces of obesity-induced gliosis and in turn, how gliosis contributes to the development and maintenance of obesity. Apart from the finding of an overall decrease in GFAP (but not Iba1) density in the DVC nuclei of animals exposed to a high-fat vs. standard diet, we did not observe any diet-induced changes in gliosis in the arcuate nucleus of the hypothalamus, a key target of leptin in energy homeostasis^25,26^. Our result is inconsistent with previous rodent (often murine) studies describing an association between a high-fat diet, increased gliosis in the hypothalamus, and the etiological development of obesity^17–19^. One possible explanation for this discrepancy is that the vast majority of these aforementioned studies were carried out in mice and therefore the HFD-induced gliosis, or lack of, may be species-dependent. Collectively, published studies reveal that gliosis occurs dynamically across time and does not persist throughout the entire duration of HFD exposure^17,19,22,75,76^. Future time-course analyses are required to fully assess HFD-induced gliosis in the hypothalamus and DVC. Nonetheless, we also observed that ZDF rats maintained on a standard diet exhibited an increase in astrogliosis in the DVC in tandem with attenuated microgliosis in the arcuate nucleus of the hypothalamus. These collective findings, although in stark contrast to previous literature, uncover a novel divergence in the leptin responses and function of glia between the DVC and hypothalamus. Whereas leptin appears to act on glia to enhance neurotransmission in the hypothalamus, leptin action in the DVC may serve to decrease neuronal output in a manner that facilitates the appetite suppressant activity of leptin. In the absence or complete dysregulation of LepR signaling, the excitatory output from the hypothalamus and inhibition of output from the DVC are lost, resulting in a loss of leptin-associated inhibitory control of food intake and weight gain.

The present study had several limitations. One caveat is that HFD-induced obese rats are hyperglycemic but not diabetic, whereas the ZDF animals display a full type II diabetic phenotype, raising the possibility that hyperglycemia and insulin resistance may contribute to the observed effect on astrocytes. Our immunohistochemical analyses of gliosis does not provide a refined characterization of the effects of diet on glia cells. Future analyses on the diet-induced effects on gliogenesis, morphological changes to glia processes, glia activation, and associations with neuronal synapses are required. Second, we relied on historic literature examining the hypothalamic leptin-astrocyte interplay^11,13–15^. It is entirely possible that each of the previous reports may have some specific detailed methodological limitations in the IHC quantifications performed.

Regardless of these limitations, the current data strongly suggest that the field would benefit from broadening our understanding of divergent astrocyte responses in different brain nuclei to obesity and alterations in leptin signaling if we are to understand fully the role that astrocytes are playing in energy balance regulation.

In conclusion, LepR is functionally expressed on astrocytes of the DVC and exert a different functional role in the DVC than it does in the hypothalamus. Unlike hypothalamic astrocytes, DVC astrocytes may be particularly vulnerable to the absence of leptin signaling. Future efforts should focus on elucidation of the neural pathways supported by the divergent functional roles of DVC vs. hypothalamic glia, as well as examine the contribution of leptin-astrocyte signaling in other CNS nuclei of relevance to energy balance control. Collectively, current findings uncover a novel role for DVC astrocytes in mediating the actions of leptin.

## Supplementary information

Supplemental Legends

Supplemental Figure 1

Supplemental Figure 2

Supplemental Figure 3

Supplemental Video 1

Supplemental Video 2

Supplemental Video 3
