# Supplementary material for: Dorsal vagal complex and hypothalamic glia differentially respond to leptin and energy balance dysregulation
Source: Transl Psychiatry. 2020 Mar 9;10:90. doi: 10.1038/s41398-020-0767-0 (PMC7062837; doi:10.1038/s41398-020-0767-0)
Supplement: Supplementary file 1 — Supplemental Legends [file 41398_2020_767_MOESM1_ESM.docx]

**Supplemental Figure 1: Synthesis of Cy5-labeled leptin** (**A**) RP-HPLC of Cy5-labeled leptin (Cy5-Lep) indicating ≥ 98% purity. (**B**) SDS-PAGE of Cy5-Lep (lanes 1 and 2) showing a 15–20 kDa product mass. (**C**) Electronic absorption spectrum of Cy5_lep with observed maxima at 651 nm. (**D**) Excitation and emission spectra of Cy5-Lep at 651 nm and 669 nm, respectively.

**Supplemental Figure 2: HFD-fed animals weighed significantly more than chow rats throughout behavioral experiments.** Experiment carried out in a counter-balanced, within-subject design. Body weights were compared at the start and end of experimental treatments. Data represented as mean + SEM, analyzed using unpaired t-test, ****p<0.0001 vs Chow, ***p<0.001 vs Chow; Chow n=11, HFD n=8.

**Supplemental Figure 3: Metabolic characterization of Zucker diabetic fatty rat.** (**A**) Body weight and (**C**) food intake recorded every 48h. (**B**) No significant difference in 48h change in body weight between wt and ZDF. (**D**) Resting blood glucose levels recorded weekly. Data represented as Mean + SEM, analyzed using multiple t-test, ****p<0.0001 compared to wt; n=5/genotype.

**Supplemental Video 1**: Representative three-dimensional rotational video of fluorescent *in situ* hybridization in the lateral NTS of the 40x image depicted in Figure 2B. Presence of the Lep-R (yellow) observed on both astrocytes (ALDH1L1; cyan) and neurons (RbFox3; magenta). Counterstained with DAPI (blue). This video is taken from a z-stack (0.99 μm step) collected from the lateral NTS at the level of the area postrema with the 40x oil-immersion objective.

**Supplemental Video 2**: Astrocyte and neuronal response to leptin in chow-maintained rat. Visible frame contains a mixed field of astrocytes and neurons in the medial NTS. Three-minute video compressed into 2 seconds.

**Supplemental Video 3**: Astrocyte and neuronal response to leptin in HFD-maintained rat. Visible frame contains a mixed field of astrocytes and neurons in the medial NTS. Three-minute video compressed into 2 seconds.
