# Supplementary material for: Dorsal vagal complex and hypothalamic glia differentially respond to leptin and energy balance dysregulation
Source: Transl Psychiatry. 2020 Mar 9;10:90. doi: 10.1038/s41398-020-0767-0 (PMC7062837; doi:10.1038/s41398-020-0767-0)
Supplement: Supplementary file 2 — Supplemental Figure 1 [file 41398_2020_767_MOESM2_ESM.pdf]

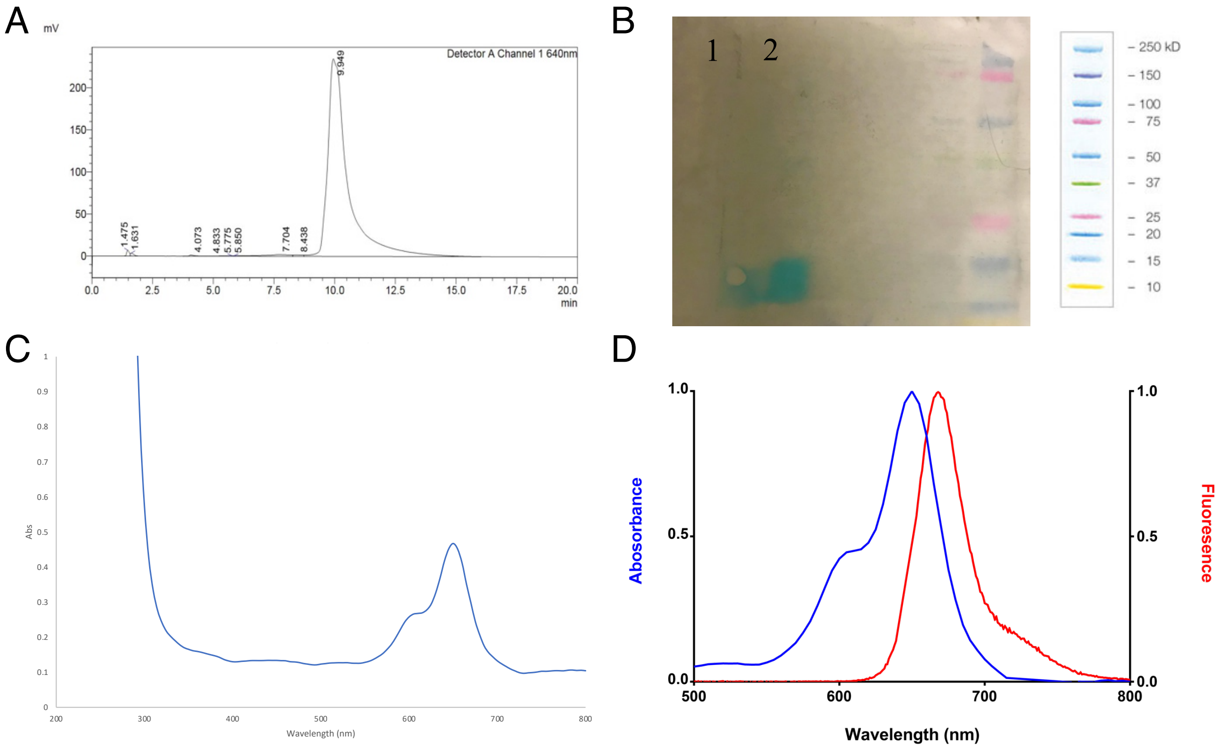

Supplemental Figure 1: Synthesis of Cy5-labeled leptin (**A**) RP-HPLC of Cy5-labeled leptin (Cy5-Lep) indicating ≥ 98% purity. (**B**) SDS-PAGE of Cy5-Lep (lanes 1 and 2) showing a 15–20 kDa product mass. (**C**) Electronic absorption spectrum of Cy5_lep with observed maxima at 651 nm. (**D**) Excitation and emission spectra of Cy5-Lep at 651 nm and 669 nm, respectively.
