# Supplementary material for: Dorsal vagal complex and hypothalamic glia differentially respond to leptin and energy balance dysregulation
Source: Transl Psychiatry. 2020 Mar 9;10:90. doi: 10.1038/s41398-020-0767-0 (PMC7062837; doi:10.1038/s41398-020-0767-0)
Supplement: Supplementary file 3 — Supplemental Figure 2 [file 41398_2020_767_MOESM3_ESM.pdf]

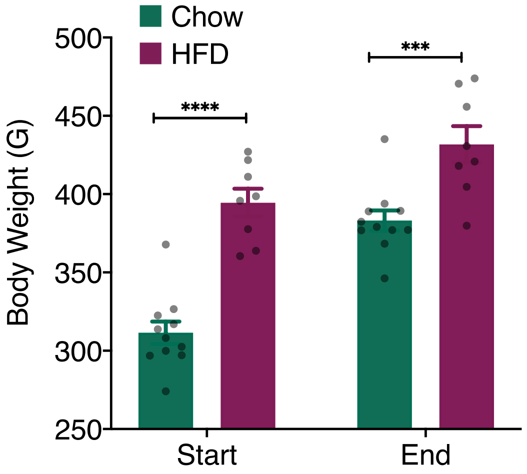

Supplemental Figure 2: HFD-fed animals weighed significantly more than chow rats throughout behavioral experiments. Experiment carried out in a counter-balanced, within-subject design. Body weights were compared at the start and end of experimental treatments. Data represented as mean $\pm$ SEM, analyzed using unpaired t-test, ****$p<0.0001$ vs Chow, ***$p<0.001$ vs Chow; Chow n=11, HFD n=8.
