# Supplementary material for: Dorsal vagal complex and hypothalamic glia differentially respond to leptin and energy balance dysregulation
Source: Transl Psychiatry. 2020 Mar 9;10:90. doi: 10.1038/s41398-020-0767-0 (PMC7062837; doi:10.1038/s41398-020-0767-0)
Supplement: Supplementary file 4 — Supplemental Figure 3 [file 41398_2020_767_MOESM4_ESM.pdf]

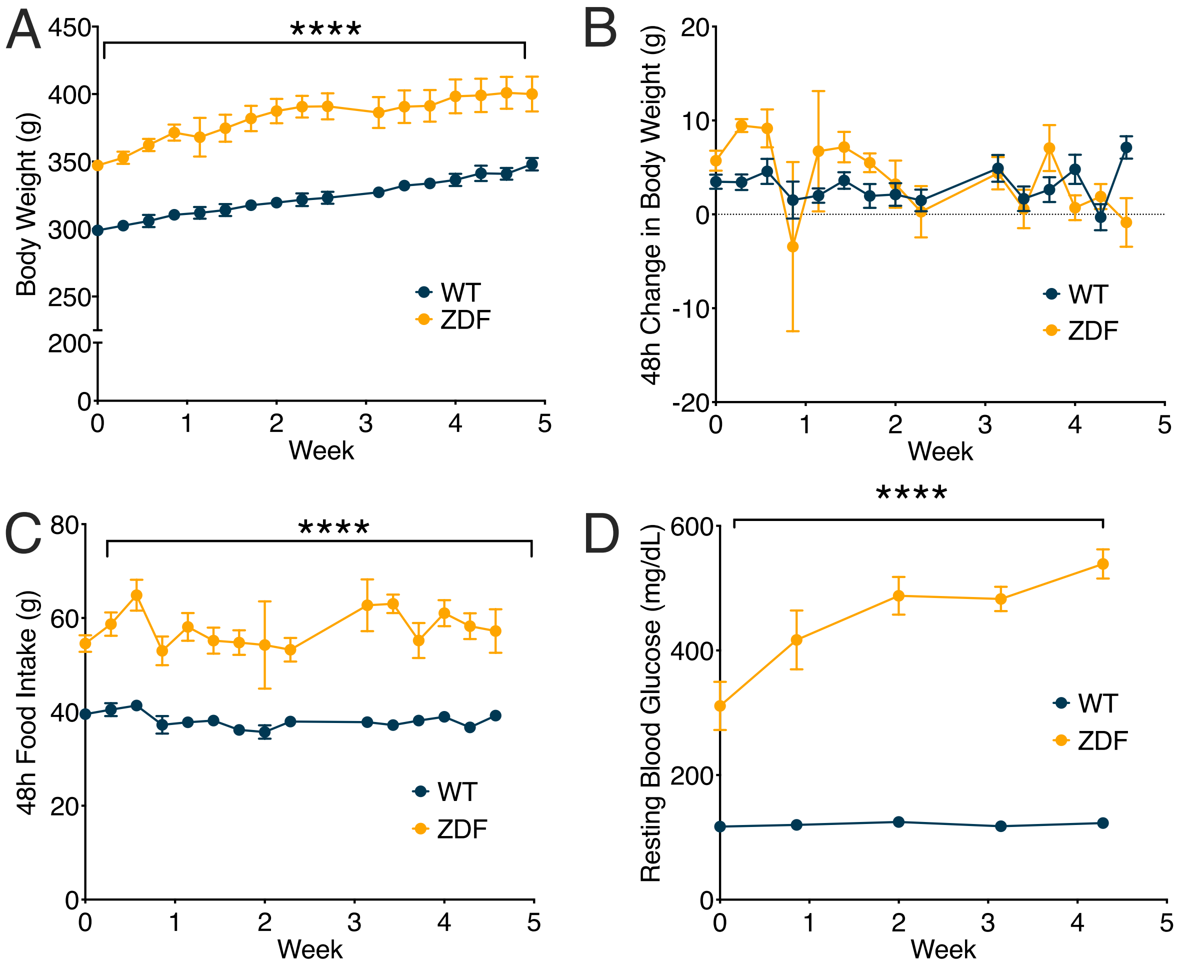

Supplemental Figure 3: Metabolic characterization of Zucker diabetic fatty rat. (**A**) Body weight and (**C**) food intake recorded every 48h. (**B**) No significant difference in 48h change in body weight between wt and ZDF. (**D**) Resting blood glucose levels recorded weekly. Data represented as Mean $\pm$ SEM, analyzed using multiple t-test, ****p<0.0001 compared to wt; n=5/genotype.
